# Hydrogen-Rich Syngas Production from Gasification and Pyrolysis of Solar Dried Sewage Sludge: Experimental and Modeling Investigations

**DOI:** 10.1155/2017/7831470

**Published:** 2017-08-09

**Authors:** Aïda Ben Hassen Trabelsi, Amina Ghrib, Kaouther Zaafouri, Athar Friaa, Aymen Ouerghi, Slim Naoui, Habib Belayouni

**Affiliations:** ^1^Laboratory of Wind Energy Control and Waste Energy Recovery (LMEEVED), Research and Technology Centre of Energy (CRTEn), Borj-Cedria Technopark, BP 95, 2050 Hammam-Lif, Tunisia; ^2^Department of Geology, Faculty of Sciences of Tunis, University of Tunis El Manar, Tunis, Tunisia; ^3^Laboratory of Microbial Ecology and Technology (LETMi), The National Institute of Applied Sciences and Technology (INSAT), Carthage University, 2 Boulevard de la Terre, BP 676, 1080 Tunis, Tunisia

## Abstract

Solar dried sewage sludge (SS) conversion by pyrolysis and gasification processes has been performed, separately, using two laboratory-scale reactors, a fixed-bed pyrolyzer and a downdraft gasifier, to produce mainly hydrogen-rich syngas. Prior to SS conversion, solar drying has been conducted in order to reduce moisture content (up to 10%). SS characterization reveals that these biosolids could be appropriate materials for gaseous products production. The released gases from SS pyrolysis and gasification present relatively high heating values (up to 9.96 MJ/kg for pyrolysis and 8.02  9.96 MJ/kg for gasification) due to their high contents of H_2_ (up to 11 and 7 wt%, resp.) and CH_4_ (up to 17 and 5 wt%, resp.). The yields of combustible gases (H_2_ and CH_4_) show further increase with pyrolysis. Stoichiometric models of both pyrolysis and gasification reactions were determined based on the global biomass formula, C_α_H_β_O_γ_N_δ_S_ε_, in order to assist in the products yields optimization.

## 1. Introduction

In Tunisia, since 1974, date of implementation of the first wastewater treatment plant (WWTP), urban wastewater treatment operations generate increasingly large quantities of sewage sludge (SS). According to the National Office for Sanitation “ONAS,” the generation of humid SS reached about 2 million tons/year in 2009 and it was estimated at 2.5 million tons/year in 2016. Despite the large quantities of produced SS, no specific handling procedure has been adopted until now in Tunisia. Actually, the greater part of generated SS (around 76%) has always been stockpiled on WWTP (35%) and on ONAS sites (41%) and only 24% was landfilled in municipal discharges to limit it negative influence on environment. In view of the current situation of SS treatment in Tunisia and with the increasing awareness regarding harmful characters of these wastes for environment and human health, valuable methods of SS management are expected urgently. Regarding the chemical composition of urban SS, which consists of heterogeneous mixture of nontoxic organic matter and inorganic compounds [1], the energetic valorization of these residues constitutes an alternative way to convert such carbonaceous materials into higher value-added products (biofuels, heat, and electricity). In this regard, to recover energy from SS, various treatment options (anaerobic digestion, incineration with energy recovery; gasification and pyrolysis, supercritical oxidation; thermal hydrolysis,* etc*.) have been advanced by [2, 3]. In Tunisia, the anaerobic digestion is the most advanced practice for energy recovery from SS and it is already applied in practice in some wastewater treatment plants such as Chotrana Station (around Tunis). However, the thermochemical conversion of SS, especially via gasification and pyrolysis processes, is still undeveloped, in Tunisia, even at the R&D stage. Thus, in order to advance the SS thermal processing field in Tunisia, the present work can be viewed as a feasibility assessment of these innovative applications (pyrolysis and gasification) to Tunisian urban SS. In fact, these thermochemical processes have been investigated in the literature for several biomass species and organic materials such as for Municipal Solid Wastes “MSW” [4], plastic [5], waste tyres [6], paper [7], feathers wastes [8], and lignocellulosic residues [9, 10]. Concerning their application to SS, some works have been published in recent times. The major parts of these works focused on the advance of new thermochemical technologies such as microwave assisted pyrolysis and catalytic pyrolysis [11, 12]. Other studies were centred on the possibility of converting SS into solid biochar valuable as fertilizers or as adsorbents via pyrolysis [13] but the produced sludge based adsorbents did not yield high surface areas due to the high inorganic content of sludge [14]. In order to improve the quality of the activated material for use as adsorbent, [15] proposed two sequential steps of washing by using first a solution of HCl (in order to remove part of the ashes) and then a solution of Na_2_CO_3_ (to extract most of the silica remaining). The other parts focused on the production of liquid biofuels (bio-oil) from SS [16, 17]. However, the use of SS to produce gaseous products using thermochemical processes has received little attention in the literature [18].

On behalf of these previous works, a rigorous comparative study should be carried to evaluate the potential of hydrogen-rich gas production from Tunisian SS using two different thermochemical processes (pyrolysis and gasification). In the present work, the production of solids, oils, and gas from Tunisian SS at high temperatures using pyrolysis was examined. The results were compared with those obtained with gasification.

It is important to distinguish pyrolysis from gasification. The main difference between pyrolysis and gasification is the absence of a gasifying agent in the case of pyrolysis. Pyrolysis is a thermal degradation of organic compounds, at a range of temperatures from 300 to 900°C, under oxygen-deficient circumstances to produce various forms of products (a liquid fraction called bio-oil, a solid fraction called biochar, and a gaseous fraction called synthetic gas or syngas), whereas gasification is a thermal cracking of solid carbonaceous material into a combustible gas mixture (syngas), mainly made up of dihydrogen (H_2_), carbon monoxide (CO), carbon dioxide (CO_2_), and methane (CH_4_) and other gases with some byproducts (solid char or slag, oils, and water). The produced syngas' chemical composition and properties are largely affected by the operational conditions throughout pyrolysis and gasification (reactor temperature, residence time, pressure, gasifying agent nature and proportion,* etc.*), by the reactor geometry, and by the feedstock characteristics (and mainly humidity) [7]. The produced syngas from pyrolysis and gasification can be applied: to run internal combustion engines to generate electricity and as substitute for fuel oils in direct heat application [19], to produce chemicals in industries [20], and to power hydrogen fuel cells [21]. Furthermore, pyrolysis and gasification processes have great flexibility in the choice of feedstocks but the major constraint of their application for SS conversion remains in the high moisture content of these wastes (varying from a few percentages to more than 95% [3]) and the occurrence of inorganic pollutants such as silicates, aluminates, and calcium and magnesium within SS. The high moisture content of SS can generate many problems related, namely, to energy consumption of the pyrolysis reactor and to the production of liquid fraction with high water content (and thus extra cost for purifying the products). In order to avoid such problems, in this study, SS solar drying was performed in order to reach a humidity content around 10 wt% [22].

Since pyrolysis and gasification are complex chemical mechanisms, which incorporate several operational and environmental challenges of carbon-based feedstock, many researchers have developed models of various types and degrees of complexity in order to make the comprehension of physical and chemical mechanisms inside the reactors easier and assist in the yield optimization. Actually, several models have been employed to simulate these thermochemical processes at different scales, namely, particle level, multiphase reacting flow, product distribution and reactor performance, and process integration and control. For pyrolysis, kinetic models, particle models, and reactor models have been proposed [23], and for gasification, equilibrium models, combined transport and kinetic models, CFD models, ANNs model, and Aspen Plus® models have been well discussed [22]. Numerous software and numerical interfaces have been employed for mathematical modeling of thermochemical processes, for example, gPROMS (Process Systems Enterprise); ANSYS Fluent®, PHOENICS®; CFD2000®; STATISTICA Neural Networks (SNN) (StatSoft®) software; Aspen Plus; and MATLAB® [22].

The present work has been undertaken to investigate the potential of gaseous biofuels production from Tunisian SS through thermochemical processes and thus to examine the main differences between the gasification and pyrolysis reactions, behaviours, and products, with special focus on the evolution of syngas composition, liquid bio-oil or tars characteristics, and solid residue properties. The final objective of this study is to initiate the development of the SS thermochemical technologies for SS handling in Tunisia, since the use of this type of waste may represent a valid energy source.

## 2. Materials and Methods

### 2.1. Materials

The dewatered SS samples were collected from “Rades Central Wastewater Treatment Plant” near Tunis (Tunisia) and then predried in a solar dryer installed in the Research and Technology Centre of Energy (CRTEn, Tunisia) for one week until a water content around 10%, since most of the previous studies on SS pyrolysis were applied on SS with humidity between 5 and 35% [22]. The solar dried samples, rounded aggregates with particle size between 0.5 mm and 2 cm in diameter, were collected in glass bottles for analysis and for pyrolysis and gasification experiments. The solar dried materials have been characterized by ultimate analysis (CHNS-O) and proximate analyses (moisture, ash content, volatile matter content, and fixed carbon) following standardized procedures. The CHN elemental composition was determined using Perkin Elmer 2400 CHN elemental analyzer, while the sulphur content was determined via HORIBA Jobin Yvon elemental sulphur analyzer [24]. SS Higher Heating Value (HHV) was calculated using the following formula [25]:

(1) HHV=0.3491C+1.1783H+0.1005S−0.1034O−0.0151N−0.0211A MJkg.

The moisture content was determined by measuring the weight loss after drying the studied samples at 105°C for 24 h [26]. The ash content was determined by dry combustion in a muffle furnace at 550°C for 3 h [26]. The volatile matter (VM) contents were determined based on the mass loss after combustion of the samples at 900°C for 4 min [26]. The fixed carbon (FC) contents were calculated using the difference between the dry matter and the sum of the ash contents plus the VM contents. The fixed carbon (FC) content was calculated in the following equation:

(2) $$\begin{array}{c} FC=DM-\left(\;VM+Ash\;\right). \end{array}$$

The hydrogen index (expressed in mg hydrocarbons/g Total Organic Carbon; mg HC/g TOC) of predried SS was determined by Rock-Eval® pyrolysis using a model VI device (Vinci Technologies). Predried SS samples were first pyrolyzed under N_2_ up to 650°C and the amounts of gaseous products (hydrocarbons, CO_2_, and CO) were continuously measured via flame-ionization (FI) and infrared detection. The TOC is determined as the sum of pyrolyzed organic carbon and residual organic carbon, the latter being determined by combustion, after the pyrolysis phase.

FTIR spectroscopic analyses were performed using a Perkin Elmer Spectrum BX spectrophotometer, in absorbance mode, between 4000 and 400 cm^−1^, on pellets made from a mixture of predried SS/KBr. The functional groups contained in studied sample were determined based on the identification of FTIR spectra according to previously published data [1, 12, 16]. The thermogravimetric measurements were performed using a “SETSYS” (Setaram Instrumentation) thermobalance. The experimental conditions were as follows: inert atmosphere (He), temperature range between 20 and 1400°C, heating rate about 20°C/min, and sample mass around 20 mg.

### 2.2. Pyrolysis and Gasification Procedures

The pyrolysis and gasification experiments' main conditions are reported in Table 1.

#### 2.2.1. Pyrolysis Experimental Setup

**Figure 1:**
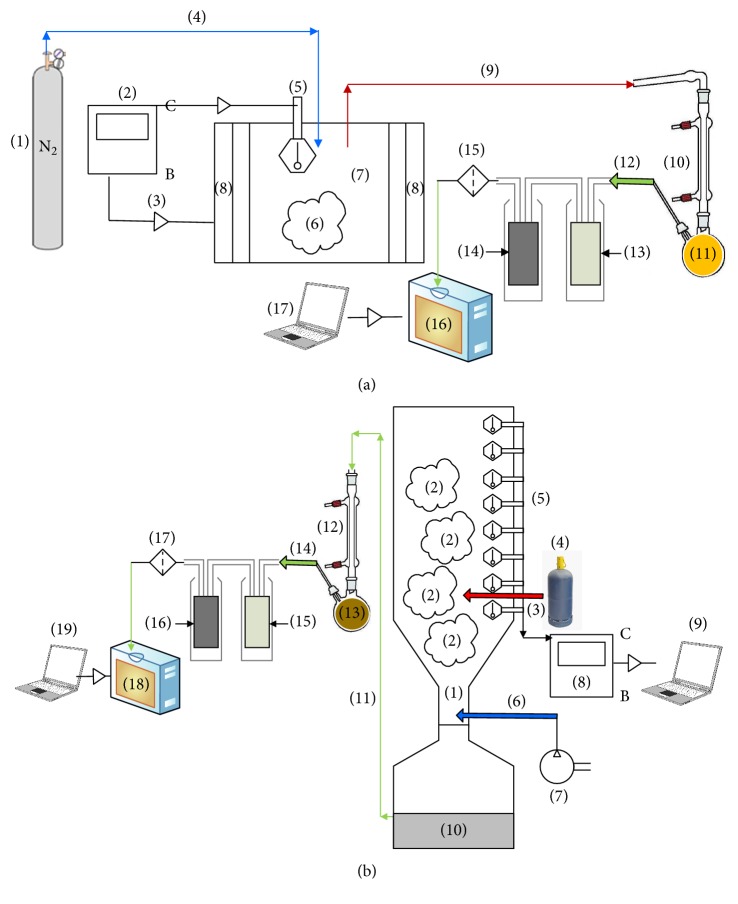
Schematics of systems used in SS pyrolysis (a) and gasification (b) experiments. (a) Pyrolysis system: (1) N_2_ gas cylinder; (2) heating control panel; (3) control transfer; (4) N_2_ inlet; (5) thermocouple; (6) sewage sludge; (7) pyrolysis reactor; (8) electrical furnace; (9) vapors outlet; (10) condensation system; (11) liquid fraction; (12) noncondensable gases; (13) gas purification system; (14) activated carbon column; (15) gas filters; (16) gas analyzer; (17) laptop for data acquisition. (b) Gasification system: (1) gasification reactor; (2) sewage sludge; (3) flame introduction; (4) gas cylinder; (5) thermocouples; (6) oxidizing agent (air) introduction; (7) air compressor; (8) temperature recorder; (9) laptop for temperature acquisition; (10) ash collector; (11) syngas outlet; (12) condensation system; (13) tars; (14) noncondensable gases; (15) gas purification system; (16) activated carbon column; (17) gas filters; (18) gas analyzer; (19) laptop for data acquisition.

The pyrolysis unit (Figure 1(a)) is a fixed-bed reactor (stainless steel made, 30 cm height, and 15 cm internal *Ø*), where the SS sample is placed in batch and then heated until the final pyrolysis temperature with an electric furnace. At the top of the reactor, there is (i) a nitrogen injection system to prevent oxygen introduction into the reactor and to guarantee an inert medium for pyrolysis reactions and (ii) a thermocouple K-type immersed inside the reactor to control the internal axial temperature. The pyrolysis vapors leave the top of the reactor and go on to the condensation system, which consists of two condensers, where a part of bio-oil is condensed and stored in a conical flask. The condensation system operates with continuous cold glycolated water (−5°C) circulation. Pyrolysis liquid products consisted of two phases, aqueous phase and organic phase, which are easily separated using decantation (Figure in Supplementary Material available online at https://doi.org/10.1155/2017/7831470). The noncondensable gases enter into a purification system constituted of many filters in series: (i) a bubbling system using distilled water to remove remaining hydrocarbons and water within noncondensable gases, (ii) an activated carbon column for retention of small particles, and (iii) low pressure gas filters to enhance the particulates and moisture removal. The chemical composition of cleaned gases is then determined using an on-line gas analyzer. The carbonaceous residue is collected in the bottom of the reactor. The pyrolysis experiments were repeated three times and the products yields presented are the mean value of three equivalent experiments. The several yields of pyrolysis products are calculated as indicated in [27].

#### 2.2.2. Gasification Experimental Setup

The gasification unit (Figure 1(b)) consists of a 50 kWth downdraft fixed-bed reactor type “Imbert” (stainless steel made, circular in cross section with 80 cm internal diameter and 120 cm height). The feeding capacity ranges from 2 to 8 kg/h. The SS sample is placed in batch in the reactor and the gasifying agent (air) is introduced continuously at the bottom part of the reactor with a steady flow rate. Axial temperature evolution is detected using a series of K-type thermocouples (8 thermocouples) and recorded automatically every 10 s by a controller computer. The produced gases pass through a quenching system for tar removal. The noncondensable gas mixture is then purified using the same gas cleaning system (water bubbler, moisture absorber, and low pressure filters) used for pyrolysis gases and described below. The chemical composition of cleaned gases is then determined using an on-line gas analyzer. The remaining solid residue, containing inorganic components, ashes, and unconverted carbon, is collected in the bottom part of the reactor after each experiment.

### 2.3. Pyrolysis and Gasification Products Characterization

The chemical composition of syngas flowing out from the pyrolysis and gasification reactors, separately, was determined using an on-line analyzer (GEIT 3160 model) equipped with (i) an Infrared detector (for analyzing CO, CO_2_, CH_4_, and light hydrocarbons (C_*n*_H_*m*_)), (ii) a Thermal Conductivity Detector (TCD) to quantify H_2_, and (iii) an electrochemical cell for O_2_ measurement. The syngas heating value is also determined by the same analyzer.

The elemental composition CHN of bio-oil released from predried SS pyrolysis was determined in the same conditions as raw materials. The fuel properties of bio-oil obtained from SS pyrolysis were measured according to the international standards used for petroleum products (density, viscosity, and water content [28]). FTIR spectroscopy was applied to bio-oil and biochar obtained from the pyrolysis of SS and to the condensable tars and solid residue obtained from the gasification of SS in the same conditions as raw materials.

### 2.4. Mathematical Modeling: Stoichiometric Model Establishment

#### 2.4.1. Raw Material Mole Numbers

According to Redfield formula reported in his study [29] in marine biology to determinate the composition of phytoplankton, the ratio of the elements does not change. The number of carbons *α* (in this case 106 according to Redfield formula) is determined in order to calculate mole number of other elements.

Let *c*_1_ and *c*_2_ be the following coefficients: *c*_1_ = %C/12 and *c*_2_ = *α*/*c*_1_.

The formula of the compound is as follows: C_*α*_H_*β*_O_*γ*_N_*δ*_S_*ε*_, with

(3) β=%H∗c21;γ=%O∗c216;δ=%N∗c214;ε=%S∗c232.1.

The mass of the compound is *m*_0_ = 12*α* + *β* + 16*γ* + 14*δ* + 32.1*ε* (in g).

Let *m*_*i*_ be the initial mass of the compound, let *p*_*i*_ be the proportion of the organic element *i*, and let *M*_*i*_ be its molar weight. The number of moles of the species *i* into the biomass is *n*_*j*_ = (*m*_*i*_*∗p*_*j*_)/*M*_*j*_ (in mol).

#### 2.4.2. Products Yields

For determined temperature and heating rate, the mass yields are named *R*_*b*_, *R*_*c*_, and *R*_*g*_, respectively, for bio-oil, biochar, and syngas. Let *m*_0_ be the initial quantity of biomass (in g); *m*_*b*_, *m*_*c*_, and *m*_*g*_ are, respectively, the masses of bio-oil, biochar, and syngas. The mass of the product* p* is *m*_*p*_ = (*R*_*p*_/100)*∗m*_0_ (in g).

#### 2.4.3. Syngas Mole Numbers

The product gases are CO, CO_2_, O_2_, H_2_, CH_4_, C_*n*_H_*m*_, and N_2_. Let *f*_*i*_ be the fraction of the gas *i*, let *M*_*i*_ be its molar mass, and let *m*_*g*_ be the mass of the product gas. The mole number of the gas *n*_*i*_ is calculated by the following equation: *n*_*i*_ = (*m*_*g*_*∗f*_*i*_)/*M*_*i*_ (in mol).

For the gasification, the masses of produced gases are calculated as follows.

*m*_*g*_ = *m*_0_*∗*(1 − (*e* + *c*)/100) (in g), with *e* being the water content of the biomass, *c* being the ash content, and *m*_0_ being the initial mass.

#### 2.4.4. Biochar Moles Numbers

Let *p*_*j*_ be the proportion of the organic element *j* and let *M*_*j*_ be its molar mass. The number of moles of the species *j* in the biochar *n*_*j*_ is *n*_*j*_ = (*m*_*c*_*∗p*_*j*_)/*M*_*j*_ (in mol).

#### 2.4.5. Organic Matter Balance

As hypothesized, N_2_ is considered as inert element in pyrolysis and will not be counted in the balance of organic matter.

## 3. Results and Discussion

### 3.1. Feed Materials Characteristics

#### 3.1.1. Elemental Composition

The elemental chemical composition, the physicochemical properties, the HHV value, and the Rock-Eval parameters of the predried SS sample are shown in Table 2. The carbon and hydrogen contents are relatively high (roughly 48 wt% and 8 wt%, resp.) and comparable to those of other SS used in pyrolysis and advanced by [16, 18, 30] and to some lignocellulosic materials [9] (Table 2). The nitrogen and sulphur contents are, respectively, about 1.71 wt% and 0.96 wt%; these contents are lower than those obtained for other SS samples (from 4 to 8 for nitrogen and from 0.6 to 7.6 wt% for sulphur; Table 2). The high nitrogen content observed in studied SS is attributed to the protein fraction contained in microorganisms used for water purification through biological degradation [31]. The oxygen content is around 10% and it is significantly lower than those of lignocellulosic residues and SS samples (approximately between 18.8 and 53.7%; Table 2).

These differences in elemental composition between SS samples cited in previous studies and those of this work could be explained by the difference in the origin and the treatments in wastewater plant [31].

Proximate analysis (Table 2) showed that SS moisture content is about 9.49% and this low humidity was reached using a solar drying technology. The purpose of the SS drying was to achieve water content less than 10%, since most of the previous studies on SS pyrolysis and gasification were applied for raw materials with humidity between 5 and 35% [1, 16, 18, 22] in order to reduce the occurrence of steam reforming reactions at high temperatures and to increase the production of hydrogen-rich gas. The amounts of ash and VM in SS are quite high (around 30.80% and 58.81%, resp.) and they are in the same range of SS used in previous pyrolysis studies and reported by other researchers [1, 18, 22, 31].

The Rock-Eval hydrogen index (HI) is around 476 mg of hydrocarbons by g of Total Organic Carbon (mg HC/g TOC), indicating a high potential of hydrogen production for the predried SS. In fact, measured usually for evaluating the source rock maturity in petroleum prospection, the HI parameter indicates the richness of studied SS in hydrogen which can be converted into gaseous hydrogenated components via pyrolysis. For the studied samples, the HI value is very high confirming them to be appropriate substrates for the production of hydrogen-rich synthesis gas such as H_2_, CH_4_, and C_*n*_H_*m*_. SS calorific value is quite high (around 25 MJ/kg) compared to those of other SS used for pyrolysis experiments (around 17 MJ/kg [18] and between 9 and 12 MJ/kg [1, 16]).

#### 3.1.2. FTIR Functional Groups Composition

**Figure 2:**
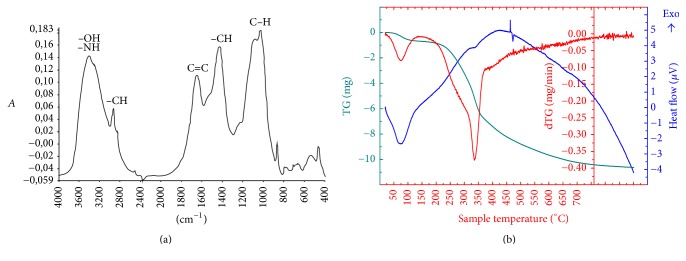
FTIR spectrum (a) and TGA/DTA diagrams (b) for solar dried SS sample.

Figure 2(a) shows the IR spectrum obtained from predried SS. The SS FTIR spectrum contains a large band between 3200 and 3600 cm^−1^ attributed to O–H stretching vibration of carboxylic and alcoholic groups. The presence of the absorption peaks between 2800 and 3000 cm^−1^ and around 1400 cm^−1^ indicates the high content of aliphatic functions originated mainly from lipid fraction contained in SS. The richness of aromatic groups is revealed by the large band at around 1640 cm^−1^ related to C=C and other little peaks around 700–900 cm^−1^ attributed to C–H in aromatic structures. The abundance of O-containing groups is observed essentially by the presence of the large absorption band around 1037 cm^−1^ assigned to C–O stretching of carbohydrates and alcohol functions and reflecting the concurrence of exopolysaccharides released by microorganisms of predried SS and/or from wastewater itself. The FTIR spectroscopy structural characterization of the predried SS reveals the high organic character of theses wastes and mainly the oxygenated and aromatic functional groups' richness. It may be concluded that these wastes are suitable feedstock for biofuels production through pyrolysis and gasification processes.

#### 3.1.3. Thermogravimetric Behaviour

The results of the thermogravimetric (TG) and differential thermal (DT) analyses of predried SS are given in Figure 2(b). The TG curve is presented in green color indicating the variation of the mass loss (expressed in mg) of studied SS under heating, whereas the curve in red color (dTG) shows the evolution of the mass loss rate (expressed in mg/min). The blue curve indicates the heat flow (expressed in *µ*V) used during experiments. The thermogravimetric analysis was applied in this study to have prior knowledge of initial and final temperatures for thermal degradation of SS. As can be observed from Figure 2(b), the predried SS thermal decomposition and thus the loss of mass took place in two big steps: the initial weight loss occurring between 50°C and 100°C and related to a drying phase (loss of physically absorbed water molecules). It corresponds to a mass loss around 4.64% of the original mass. A second strong weight loss (around 46.43% of its original mass) occurs between 200°C and 600°C with a large peak centred at 337°C and a shoulder between 220 and 320°C. This step corresponds mainly to the volatilization of volatiles in SS and thus to the degradation of main organic components contained in SS. In reality, various organic compounds decompose between 200 and 400°C: hemicelluloses, cellulose and lignins, leather, some N-containing compounds such as aliphatic amino acids, and aromatic compounds contained in plastic materials. But, due to the great heterogeneity of the studied SS chemical composition, it is difficult to attribute this decomposition to specific organic compounds. After 600°C, SS sample showed a weight loss of 3.16% of its original mass, attributed mainly to the decomposition of inorganic materials.

### 3.2. Pyrolysis and Gasification Products Distribution

**Figure 3:**
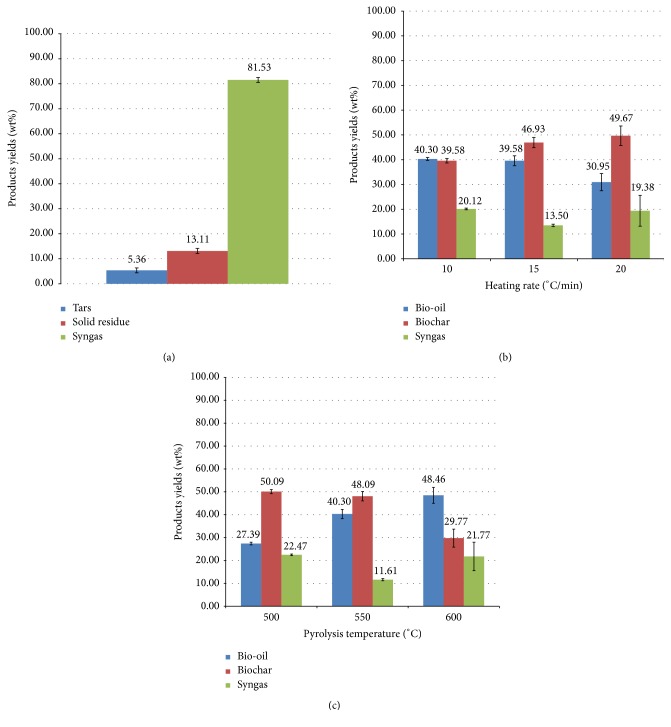
SS pyrolysis products distribution: (a) variable pyrolysis temperature and invariable heating rate, 15°C/min; (b) variable heating rate and invariable temperature, 550°C; (c) SS gasification products distribution.

Pyrolysis experimental conditions influence the products distribution and properties. Among these parameters, pyrolysis final temperature has the largest effect on the products yields. The pyrolysis temperature has been evaluated based on the thermogravimetric results and the previous studies of SS pyrolysis found in the literature.  Figure 3(a) exhibits the products (bio-oil, biochar, and syngas) distribution of SS pyrolysis at different final temperatures (500, 550, and 600°C) and at an invariable heating rate (15°C/min) and a constant cooling temperature (−5°C). As it can be seen, an increase in pyrolysis temperature (from 500 to 600°C) results in a decrease in biochar yield (from 50% to 30%) and an increase in liquid fraction yield (from 27% to 48%) due probably to the promotion of devolatilization reactions. At higher temperatures, secondary reactions involving volatiles, such as thermal cracking, are improved, leading to a reduction in the liquid yield and an increase in the gas yield. The decrease in biochar yield is possibly related also to further pyrolysis conversion and thus to a greater primary decomposition of the initial feedstock or to secondary reactions of the solid residue [13]. The syngas yield is variable; in all conditions, the pyrolysis gas amount is not very high (does not exceed 23%) and the optimum pyrolysis temperature obtained for maximum syngas production is around 500°C. By investigating the effect of reaction temperature (in the range of 450–600°C) on the yields of SS pyrolysis products, [1] obtained a maximum gas yield of 20% in dry and ash-free basis at 600°C, whereas [32] reported a maximum yield of gaseous products in the range of 15–25% at high temperature (700°C). Reference [17], by studying the influence of pyrolysis temperature on products distribution, reported that gas yield remains almost constant (around 20–22%) as the pyrolysis temperature increased from 350 to 550°C but, above 550°C, a significant increase in gas production is observed (reaching a gas yield around 32%).

Apart from temperature, heating rate also plays a significant role in products yields variation during SS pyrolysis.  Figure 3(b) shows the influence of heating rate variation on products yields distribution at an invariable final temperature (550°C) and a constant cooling temperature (−5°C). It can be seen that an increase in the heating rate leads to a decrease in the liquid fraction and to an increase in the solid fraction, while the gas fraction yield remains variable. These results are in agreement with the literature. Reference [33], using two heating rates (5°C/min and 60°C/min), demonstrated that the increase in heating rate gives an increase in liquid products yield and a decrease in biochar yield.

Regarding the gasification process, some operational problems were encountered in the measurement of products yields such as (i) tars condensation and deposits during cooling of the gasifier and at the outlet of the reactor and (ii) presence of tars in the produced syngas. Roughly, the obtained yields, given in Figure 3(c), are as follows: 82% of syngas, 13% of solid residue, and 5% of tars.

### 3.3. Gasification Reactor Temperature Distribution

**Figure 4:**
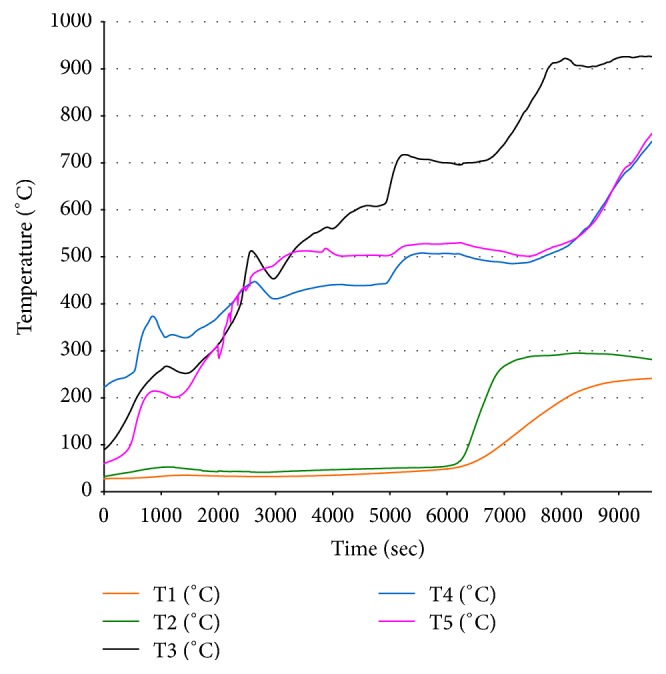
Reactor axial temperature evolution during solar dried SS gasification process.

In order to distinguish the different zones within the gasification reactor, the temperature data recorded inside the gasifier were exploited.  Figure 4 shows the inside reactor axial temperature evolution, obtained during the predried SS gasification experiences, using five active thermocouples (from T1 to T5). The temperature curves recorded by thermocouples T1 (orange color) and T2 (green color) show a regular temperature variation around 50°C until 100 min from the beginning of the gasification experiment. After that, the temperature increased slowly for T1 and suddenly for T2 to reach 240°C and 290°C, respectively, which were maintained until the end of the experiment. This corresponds to the drying zone in the reactor. In this drying zone, moisture content of the biomass is reduced considerably. The temperature variations recorded by thermocouples T3 (pink color) and T4 (blue color) show a regular evolution from room temperature to a maximum of 520°C and by the end of the experiment to 750°C as maximum temperature indicating the pyrolysis phase. In this pyrolysis stage, the thermal cracking of biomass occurs in the absence of oxygen and solid residue (charcoal) and volatile vapors (containing carbon monoxide, hydrogen, carbon dioxide, and hydrocarbon gases) are released [22]. Thermocouple T5 (black color) recorded temperatures in the gasification zone as it reaches rapidly high temperature above 900°C. The gasification process of predried SS samples started at moderate temperatures (between 600 and 700°C) and achieves high temperatures (above 900°C) at the end of the gasification experiment. In this reduction area, several reactions occur and the temperature ranges between 800 and 1000°C. These reactions are mostly endothermic in nature. The distribution of the gasifier axial temperature enables us to follow different mechanisms occurring throughout the gasification process and thus to delineate the different zones within the reactor such us drying, pyrolysis, combustion, and gasification. Furthermore, the temperature evolution shows that the predried SS gasification reactions take place at high temperature (above 900°C). In this gasification area, several reduction reactions occur. The main reactions in this zone as described by [22] are as follows.

*Water-Gas Reaction*

(4) $$\begin{array}{c} C+H_{2}O⟶CO+H_{2}\;\Delta H=131.4\text{ kJ}/mol \end{array}$$

*Boudouard Reaction*

(5) $$\begin{array}{c} C+CO_{2}⟶2CO\;\Delta H=172.6\text{ kJ}/mol \end{array}$$

*Shift Reaction*

(6) $$\begin{array}{c} CO_{2}+H_{2}⟶CO+H_{2}O\;\Delta H=42\text{ kJ}/\text{mol} \end{array}$$

*Methane Reaction*

(7) $$\begin{array}{c} C+2H_{2}⟶CH_{4}\;\Delta H=75\text{ kJ}/\text{mol} \end{array}$$

In total, the fixed-bed downdraft gasifier is recommended equipment to study the gasification reactions and mechanisms at laboratory scale and to produce clean gas but the main disadvantages of this type of gasifier are its low thermal efficiency and the great difficulties related to handling biomass with high moisture and ash contents [22].

### 3.4. Pyrolysis and Gasification Products Characterization

#### 3.4.1. Syngas Composition

**Figure 5:**
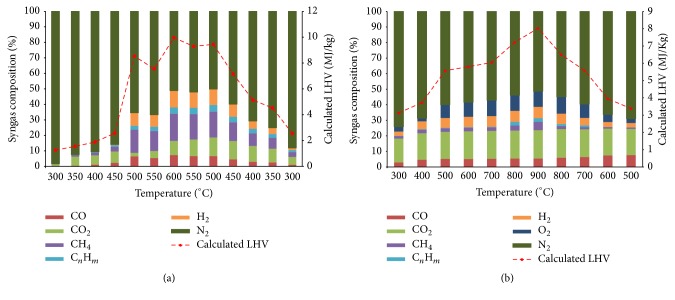
Syngas chemical composition and LHV evolution during (a) SS pyrolysis process (600°C and 20°C min^−1^) and (b) SS gasification process.

(*1) Pyrolysis Syngas Composition. The syngas* chemical composition obtained throughout SS pyrolysis is shown in Figure 5(a). The major gaseous products obtained from SS pyrolysis are CH_4_ (up to 17.62%), CO_2_ (up to 12.03%), H_2_ (up to 10.85%), CO (up to 7.26%), and C_*n*_H_*m*_ (up to 4.30%). From Figure 5(a), it could be roughly seen that the variation of several produced gases is almost constant with a high release at the beginning of the experiment (from 300°C) to achieve maximum of combustible gas production at 600°C and finally a gradual decrease marking the end of the pyrolysis reactions. Among combustible gases, CH_4_ and H_2_ are found to be the most abundant compounds of the produced gases from SS pyrolysis. The H_2_ high content in the syngas mixture produced by pyrolysis could be explained by the catalytic effect of the minerals contained in studied SS (ash content around 30.80%). This catalytic effect in SS pyrolysis was highlighted by many authors [12, 16] who studied the correlation between the H_2_ percentage produced by pyrolysis of SS and the SS ash content and demonstrated that the catalytic effect of the ash in the dehydrogenation reactions enhances the H_2_ production, whereas the high H_2_ production is often correlated directly to the high ash content of SS, which have a catalytic effect during thermal cracking and mainly in the dehydrogenation reactions [12, 16]. Moreover, the formation of CO and CO_2_ could be attributed to the decomposition of oxygenated groups such as decarboxylation and decarbonylation [32]. Other works that were conducted on SS pyrolysis have reported similar gas compositions. Reference [12] presented a syngas composition produced from SS pyrolysis with a high yield (between 45 and 66%) of CO and H_2_ and reported that the gas from the conventional oven is much richer in hydrocarbons (25%) than that from the microwave ovens (6–11%). According to [1] that studied SS flash pyrolysis in a conical spouted bed reactor at 600°C, the gas mixture composition is as follows: 35% of CO_2_, 23% of CO, 23% of H_2_, and 24% of C_1_–C_4_. Reference [16] obtained with the pyrolysis of three SS samples a gas mixture containing up to 60% of CO_2_, 34% of CO, 28% of H_2_, 11% of CH_4_, and 2-3% of C_*n*_H_*m*_.

The calorific value (LHV) of the produced gas mixture obtained under the pyrolysis conditions used in the present study reaches a maximum of 9.96 MJ/kg, indicating a high content of combustible gases in this gaseous mixture. This relatively elevated heating value of the gas mixture can be valorized as an alternative source of heating for the pyrolysis reactor. The obtained heating values of gases are in the same range as those reported by previous studies. In fact, the heating values of gases from conventional pyrolysis were 13.0–14.0 MJ/m^3^, whereas with the microwave method the heating values ranged from 6.6 to 8.6 MJ/m^3^ [11]. Reference [33] that studied the pyrolysis of SS reported that the released gases have a maximum heating value around 25 MJ/m^3^.

(*2) Gasification Syngas Composition. *The syngas chemical composition analysis throughout the gasification experiment (Figure 5(b)) reveals that the obtained syngas is a mixture of the following main components: CO_2_ (up to 18.43%), CO (up to 7.6%), H_2_ (up to 7.32%), CH_4_ (up to 5.36 %), and C_*n*_H_*m*_ (up to 2.36%). As can be observed in Figure 5(b), when the reactor temperature increased, the CO and CO_2_ concentrations increased due to decarbonylation and decarboxylation reactions at high temperatures. H_2_ concentration increased also with the temperature increase with a maximum around 7.32 wt% at nearly 900°C and this is due to not only the cracking reactions but also dehydrogenation reactions promoted by the catalytic effect of the inorganic fraction [1, 16]. CH_4_ and C_*n*_H_*m*_ showed the same trend with a slight variation from the beginning. These drawn data are in agreement with those described by [2], where a typical gasification gaseous mixture is characterized by a high hydrogen content (H_2_: 8.89–11.17 vol%). It contains also other combustible compounds, including CO (6.28–10.77 vol%), CH_4_ (1.26–2.09 vol%), carbon dioxide, and some light hydrocarbons. Similar observations have been advanced by [17] that studied SS gasification using downdraft reactor and indicated that produced syngas is composed mainly of combustible gases such as H_2_ (8.80–11.15 vol%), CO (6.31–10.63 vol%), CH_4_ (1.25–2.07 vol%), and C_*n*_H_*m*_ (0.76–1.10 vol%) and noncombustible gases such as N_2_ and CO_2_.

The heating value of the syngas obtained under the gasification conditions used in the present study reaches a maximum of 8.02 MJ/kg. This calorific value is higher than those reported for SS pyrolysis. Reference [18] reported gross heating values ranging from 4.87 to 5.55 MJ/kg for syngas produced from SS gasification in a downdraft gasifier, whereas [34] advanced LHV values ranging between 4.15 and 7.09 MJ/kg for syngas produced from two types of SS.

#### 3.4.2. Bio-Oil and Biochar Properties

The bio-oil elemental composition is as follows: C: 56.59 wt%; H: 8.24 wt%; N: 3.96 wt%; S: 0.32 wt%; and O: 30.89 wt%. The obtained carbon, hydrogen, and sulphur contents are lower than those found by other authors from pyrolysis of SS. Thus, [34] obtained, for bio-oils produced from conventional pyrolysis of SS, carbon content between 70.9 and 84.5 wt%, hydrogen content between 10.3 and 12.4 wt%, and sulphur content between 0.2 and 0.6 wt%. Reference [33] reported for organic fraction of SS pyrolysis liquids between 49.4 and 62.6 wt% for carbon and between 9.8 wt% and 11.5 wt% for hydrogen; nitrogen and sulphur contents are almost invariable [33, 35]. The knowledge of elemental composition (mainly carbon, hydrogen, and sulphur contents) is necessary for potential utilization of these bio-oils as biofuels or for further processing to petrochemicals or biodiesel.

The bio-oil density at 15°C, kinematic viscosity, and water contents of both organic and aqueous fractions from SS pyrolysis are reported in Table 3. The organic fraction density around 0.9743 Kg/m^3^ is quite higher than those required by the Tunisian standard fuels (between 820 and 860 kg/m^3^) and also higher than those of similar bio-oils obtained from different feedstocks [27]. The organic fraction viscosity is around 6.3 cSt, while the aqueous fraction one is about 10.6 cSt; these values are considerably higher than those of the Tunisian standard fuels (4.5 cSt) and those of other bio-oils reported in [27]. The water content of the organic fraction is quite high (around 16%) and this may be reduced with paying attention to the decantation of the two liquid phases.

**Figure 6:**
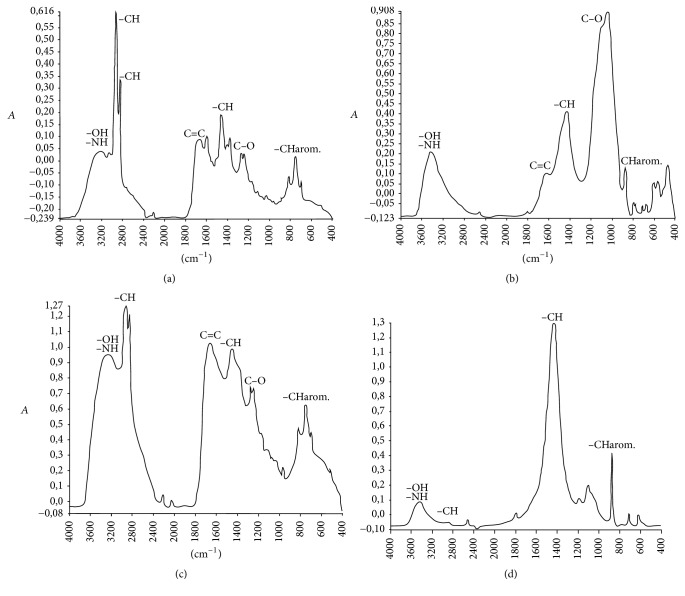
FTIR spectra of (a) bio-oil sample obtained by SS pyrolysis, (b) biochar sample obtained by SS pyrolysis, (c) tar sample obtained by SS gasification, and (d) solid residue sample obtained by SS gasification.

The FTIR spectrum of bio-oil sample obtained from SS pyrolysis is given in Figure 6(a). The liquid bio-oil seems to be very complex mixture with a high content of aliphatic groups indicated by the presence of intense peaks in the absorption region between 2800 and 3000 cm^−1^ and several peaks between 1300 and 1500 cm^−1^ imputable to aliphatic C–H deformation. The aromatic character of the bio-oil sample is marked by the peak around 1640 cm^−1^ corresponding to C=C in aromatic structures and by many little peaks in the region between 700 and 900 cm^−1^.

The biochar sample spectrum (Figure 6(b)) shows high content of O-containing functions: the band centred around 3300 cm^−1^ due to the presence of O–H groups and the high intensive band between 1000 and 1100 cm^−1^ corresponding to C–O stretching of carbohydrate and alcohol functions. The aliphatic groups' content is indicated mainly by the large band between 1300 and 1500 cm^−1^ imputable to aliphatic C–H deformation. Usually, the produced biochar obtained from SS pyrolysis could be used as biofuel (high calorific value) or as chemical adsorbent of pollutants (substitute for activated carbon) or as soil fertilizer [35]. The potential use of biochars in these three options depends on their characteristics.

#### 3.4.3. Gasification Coproducts Characterization

Figures 6(c) and 6(d) show the FTIR spectra obtained from condensable tars and solid residue produced from gasification of predried SS, respectively. The FTIR condensable tar spectrum shows the high complexity of these products. The studied tar sample is composed of aliphatic compounds and aromatic and O-containing groups with a large variety of organic compounds. The solid residue spectrum exhibits a slight absorption band between 3200 and 3600 cm^−1^ attributable to C–O stretching but a very intensive peak around 1400 cm^−1^ corresponding to aliphatic groups. We note the presence of many little peaks in the absorption region of 700–900 cm^−1^ imputable to the aromatic stretching vibrations.

### 3.5. Mathematical Modeling: Stoichiometric Model

#### 3.5.1. Pyrolysis Balance

Equation (8) of pyrolysis is

(8) $$\begin{array}{c} C_{\alpha }H_{\beta }O_{\gamma }+xH_{2}O\overset{yields}{\to }\\ yCO+zCO_{2}+tH_{2}+uCH_{4}+C_{n}H_{m}+vO_{2} \end{array}$$

*α*, *β*, and *γ* are determined by the elemental analysis.

*t*, *u*, *y*, and *z* are the number of moles of gas species calculated in Section 3.5.3.

*n*_C_, *n*_O_, and *n*_H_ are calculated in Section 3.5.1.

*x*, “the number of moles of H_2_O,” is the difference between the number of moles of oxygen in the products and that contained in biomass *γ* and it is calculated as follows:

(9) $$\begin{array}{c} x=y+2z+2v+n_{O}-\gamma \;\;\left(\;in\;\;mol\;\right). \end{array}$$

*n* is the difference between the number of moles of carbon in the initial biomass and that contained in the obtained products and it is calculated as follows:

(10) $$\begin{array}{c} n=\alpha -n_{C}-y-z-u\;\;\left(\;\text{in mol}\;\right). \end{array}$$

*m* is the difference between the number of moles of hydrogen in the reactant and that in the products and is calculated as follows:

(11) $$\begin{array}{c} m=\beta +2x-2t-4u\;\;\left(\;\text{in mol}\;\right). \end{array}$$

#### 3.5.2. Gasification Balance

The reaction of gasification is described as follows:

(12) $$\begin{array}{c} C_{\alpha }H_{\beta }O_{\gamma }+x\left(\;O_{2}+3.67N_{2}\;\right)\overset{yields}{\to }\\ yCO+zCO_{2}+tH_{2}+uCH_{4}+C_{n}H_{m}+pN_{2}+qO_{2} \end{array}$$

*α*, *β*, *γ*, and *δ* are determined by the elemental analysis.

*t*, *u*, *y*, *z*, *p*, and *q* are the number of moles of the gas species calculated in Section 3.5.3.

*x*, “the number of moles of air,” is the difference between the number of moles of oxygen in the products and that contained in the biomass and is calculated as follows:

(13) $$\begin{array}{c} 2x=2q+y+2z-x-\gamma \;\;\left(\;\text{in mol}\;\right). \end{array}$$

The number of moles *p* of nitrogen produced is calculated as follows:

(14) $$\begin{array}{c} p=x*3.76\;\;\left(\;\text{in mol}\;\right). \end{array}$$

*n* is the difference between the number of moles of carbon contained in the initial biomass and that contained in the products and is calculated as follows:

(15) $$\begin{array}{c} n=\alpha -y-z-u\;\;\left(\;\text{in mol}\;\right). \end{array}$$

*m* is the difference between the number of moles of dihydrogen in the reactants and that contained in the products and is calculated as follows:

(16) $$\begin{array}{c} m=\beta -2t-4u\;\;\left(\;\text{in mol}\;\right). \end{array}$$

#### 3.5.3. Calculations

Table 4 shows the raw material and syngas stoichiometric model calculations. Pyrolysis and gasification balances are, respectively, illustrated by (17) and (19).

*For Pyrolysis*

(17) $$\begin{array}{c} C_{\alpha }H_{\beta }O_{\gamma }+xH_{2}O⟶\\ yCO+z{CO}_{2}+tH_{2}+uCH_{4}+C_{n}H_{m}+v{HO}_{2} \end{array}$$

*α*, *β*, and *γ* were determined using SS elemental analyses data.

*t*, *u*, *y*, and *z* are the number of moles of gas species calculated using syngas composition analyses.

*n* and *m* were determined using biochar, bio-oil, and syngas elemental analyses data.

According to data reported in Table 4, we obtain the global SS pyrolysis formula:

(18) C106H215.56O16.73+13.26H2O⟶1.09CO+1.18CO2+22.86H2+4.62CH4+C299.09H124.7+vHO2

*For Gasification*

(19) $$\begin{array}{c} C_{\alpha }H_{\beta }O_{\gamma }+x\left(\;O_{2}+3.67N_{2}\;\right)⟶\\ yCO+zCO_{2}+tH_{2}+uCH_{4}+C_{n}H_{m}+pN_{2}+qO_{2} \end{array}$$

*α*, *β*, and *γ* were determined using SS elemental analyses data.

*t*, *u*, *y*, *z*, *p*, and *q* are number of moles of gas species calculated using syngas composition analyses.

*n* and *m* were determined using biochar, bio-oil, and syngas elemental analyses data.

According to data reported in Table 4, we obtain the global SS gasification formula:

(20) C106H215.56O16.73+1.27O2+3.67N2⟶3.2CO+6.8CO2+59.77H2+2.3CH4+C127.62H86.48+4.67N2+0.47O2

The obtained pyrolysis and gasification formulas describe average degradation of SS compounds under pyrolysis and gasification circumstances. The model calculations allow us to study the influence of process parameters and thus to predict the desired products yields.

## 4. Conclusions

In this study, pyrolysis and gasification of Tunisian solar dried SS were performed separately using two different laboratory-scale reactors to produce mainly hydrogen-rich synthetic gas. Through this work, we highlighted the opportunity of employment of these two processes in the conversion of Tunisian SS into combustible gases and thus the potential of solar dried SS wastes as feedstocks for syngas production. The released gases from pyrolysis (yield reaching 23 wt%) and gasification (yield reaching 82 wt%) present high calorific values (around 9.96 MJ/m^3^ for pyrolysis and 8.02 MJ/kg for gasification) due to their high contents of H_2_ (up to 10.85% and 7.32%, resp.) and CH_4_ (up to 17.62% and 5.36%, resp.). The produced syngas should be used either as a fuel or as an intermediate in the production of liquid fuels and chemicals. We suggest its reuse to supply a part of the energy needed in the heating part of the reactor in order to enhance the energy balance of the process.

Taking into account these experimental and numerical results, it can be concluded that the SS energy recovery via pyrolysis and gasification is a viable method for reducing the volume of these harmful products with producing valuable products and alternative fuels in order to reduce the consumption of fossil fuels.

In practice, for SS management policy in Tunisia, the energetic conversion using thermochemical processes is not developed. From the obtained results, the feasibility and the effectiveness of the SS thermochemical conversion were proven and thus these valorization technologies could be proposed to rationalize the disposal of SS and to offer an economical alternative, since the use of these biosolids may represent a valid energy source.

## Supplementary Material

The supplementary material shows two photos of the obtained liquid fractions from pyrolysis of pre-dried SS: the organic fraction in the left and the aqueous fraction in the right.

## Figures and Tables

**Table 1:** Pyrolysis and gasification processes conditions.

	Pyrolysis	Gasification
Raw material	Predried SS	Predried SS
Carrier gas	Nitrogen	Air
Reaction temperature (°C)	500-550-600	>900
Heating rate (°C min^−1^)	10-15-20	—
Condensation temperature (°C)	−5	−5
Reaction time (min)	~50	~160

**Table 2:** Characteristics of studied solar dried SS and others used in thermochemical conversion studies according to literature.

	Ultimate analysis (%)				
	Studied solar dried SS	Thipkhunthod et al., 2007	Tsai et al., 2009	Fonts et al., 2012	Chen et al., 2015
		Sewage sludge			Lignocellulosic biomass
C	48.21 ± 1	27.6–48.4	31.6–42.3	23.1–39.9	42.1–49.3
H	8.17 ± 0.3	6.5–7.4	4.9–6.3	3.8–5.9	5.5–6.1
O	10.15	35.3–53.7	31.8–35.6	18.8–23.5	44.2–50.9
N	1.71 ± 0.1	3.9–7.4	5.5–7.7	2.5–7.9	0.0–0.9
S	0.96 ± 0.1	1.2–7.6	0.6–1.4	0.8–1.0	0.1–0.8
	Proximate analysis (%)				
Moisture content	9.49 ± 0,3	3.2–7.6	16.0–18.0	1.5–7.1	3.6–10.3
Ash content	30.80 ± 2	43.4–71.4	24.2–44.9	22.6–52.0	0.5–7.9
Volatile matter	58.81 ± 3	25.9–52.4	39.0–54.8	38.3–66.8	71.8–83.2
Fixed carbon	0.90	2.7–6.4	—	0.8–19.7	5.7–17.4
Hydrogen index^*∗*^	476	—	—	—	—
HHV (MJ/kg)	24.82	—	—	—	—

*∗* in mg hydrocarbon/g organic carbon.

**Table 3:** Fuel properties of liquid fractions obtained from SS pyrolysis (at 550°C and 15°C/min).

	Density (15°C) (Kg/L)	Viscosity (40°C) (mm^2^/s)	Water content (%)
Organic fraction	0,9743	6,3	16
Aqueous fraction	1	10,6	25

**Table 4:** Raw material and syngas stoichiometric model calculations.

	Element	Number of moles
Raw material	Carbon	106
	Hydrogen	215.56
	Oxygen	16.73
	Nitrogen	3.22
	Sulfur	0.78

Syngas	M_g_	419.93 g
	*n*_N_2__	0.00
	*n*_O_2__	0.00
	*n*_CH_4__	4.62
	*n*_CO_	1.09
	*n*_CO_2__	1.18
	*n*_H_2__	22.86
