# Supplementary material for: Hydrogen-Rich Syngas Production from Gasification and Pyrolysis of Solar Dried Sewage Sludge: Experimental and Modeling Investigations
Source: Biomed Res Int. 2017 Aug 9;2017:7831470. doi: 10.1155/2017/7831470 (PMC5569640; doi:10.1155/2017/7831470)
Supplement: Supplementary file 1 — The supplementary material shows two photos of the obtained liquid fractions from pyrolysis of pre-dried SS: the organic fraction in the left and the aqueous fraction in the right. [file 7831470.f1.docx]

**E-** **supplement**


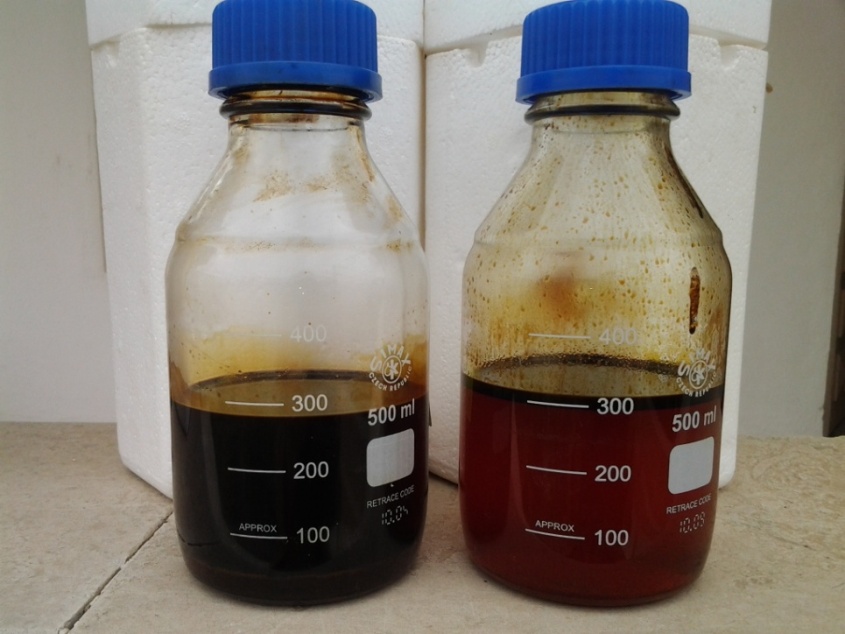


This photo shows the two obtained liquid fraction: organic fraction in the left and aqueous fraction in the right.
